# Ash Tea as the Remedy for the Bite of a Rattlesnake

**Published:** 1859-01

**Authors:** 


*Ash Tea as the Remedy for the Bite of a Rattlesnake.*—Dr. George S. Blackie:—Dear Sir, An old Tennessee friend of mine writes to me to know if I were ever called to attend a patient suffering from the bite of a rattlesnake, or any other poisonous reptile; if so, to know my treatment, and the result of that treatment, as he is in misery through fear of being bitten by one. As he is a subscriber to your valuable Journal, I send my answer to you for publication, if you deem it worthy of filling a page for you.

My answer is, that I have treated two patients bitten by rattlesnakes, and one by a spreading adder. In every case the treatment was the same, with like results. The first was a negro woman. While binding fodder late one evening, she was bitten on the fleshy part of the arm. I gave her about one pint of ash tea, prepared by taking a handful of the inner bark of the ash, adding one quart of water and boiling down to a pint. I do not give it all at once, but about half a gill every twenty minutes. As soon as the patient has taken about two portions, he will break out into a profuse perspiration. I also appled a poultice of the bark to the bitten part. On the following day the negro went to the field as usual. Case second was treated in the same manner with the same results.

Case third was Mr. N., who had hid a bottle of whisky behind the gate post, and wanting a dram about noon, reached his hand through the crack of the fence for it, and was bitten by this rusty old adder, who was guarding the bottle. The same treatment was adopted, but the patient was three days in recovering.

I am satisfied that the tea prepared from the ash bark is an effectual and safe antidote for the poisonous bite of such serpents as frequent this part of the country. The tea thus prepared is as bitter as quinine itself. Being satisfied that it is a certain and speedy antidote, I never pretend to do anything else, always applying a poultice of the bark to the bitten part. Why the remedy has not been more generally used, I cannot say. It was a new thing to me in the spring of 1854.

I draw my conclusions from the following facts: 1st, That after using the ash tea, a moderately large dram will produce intoxication as if nothing had happened. 2d, That a rattlesnake will not snap or bite at an ash pole. You may torment him with a pole of any other kind of wood, until he is entirely mad, then try the ash pole, and he will coil himself up, and no effort on your part can induce him to strike a stick of ash wood. How ash tea ever came into use as an antidote for the bite of a poisonous serpent I know not. I have the history of its origin from an old Frenchman, who follows hunting and trapping. He says that a hunter was once bitten by a rattlesnake, and the effect produced by the bite was sickness and blindness, and the hunter becoming hungry commenced to gnaw everything that same in his way; among the rest he fell up on an ash root, which entirely relieved the sickness and also the blindness. Whether his statement is entiled to any confidence or not, I am unable to say.

Yours respectfully,

—*Nashville Jour. of Med.* ARKANSAS SWAMP DOCTOR.

